# Draft Genome Sequence of *Paenibacillus polymyxa* Strain Mc5Re-14, an Antagonistic Root Endophyte of *Matricaria chamomilla*

**DOI:** 10.1128/genomeA.00861-15

**Published:** 2015-08-06

**Authors:** Martina Köberl, Richard A. White, Sabine Erschen, Tarek F. El-Arabi, Janet K. Jansson, Gabriele Berg

**Affiliations:** a Graz University of Technology, Institute of Environmental Biotechnology, Graz, Austria; b Pacific Northwest National Laboratory, Biological Sciences Division, Richland, Washington, USA; c Ain Shams University, Faculty of Agriculture, Cairo, Egypt; d Heliopolis University, Biotechnology Laboratory, Cairo, Egypt

## Abstract

*Paenibacillus polymyxa* strain Mc5Re-14 was isolated from the inner root tissue of *Matricaria chamomilla* (German chamomile). Mc5Re-14 revealed promising *in vitro* antagonistic activity against plant and opportunistic human pathogens. The 6.0-Mb draft genome reveals genes putatively involved in pathogen suppression and direct and indirect plant growth promotion.

## GENOME ANNOUNCEMENT

*Paenibacillus polymyxa* strain Mc5Re-14 was isolated in April 2010 from the endorhiza of the German chamomile *Matricaria chamomilla* L., cultivated on the organically managed Sekem farms in the northeastern desert region of Egypt (30°22′88″N 31°39′41″E) (1). The soil texture at the desert farm was classified as loamy sand, with a clay content of 4%, organic carbon content of 0.8%, and an alkaline pH of 8.4 (2). Mc5Re-14 exhibited broad-spectrum antagonism against soilborne phytopathogenic fungi (*Verticillium dahliae*, *Fusarium culmorum*, and *Rhizoctonia solani*) and nematodes (*Meloidogyne incognita*) and was also active against the opportunistic human pathogen *Escherichia coli* (3, 4). Treatment of chamomile plants with Mc5Re-14 under field conditions resulted in an elevated flavonoid content in the blossoms (5).

Genomic DNA was extracted using the MasterPure DNA purification kit (Epicentre, Madison, WI, USA), modified with additional cell disruption steps comprising mechanical shredding with glass beads in a FastPrep instrument (MP Biomedicals, Santa Ana, CA, USA) and lysozyme-based cell wall digestion. PacBio RS libraries with inserts of 8 to 12 kb were constructed and sequenced at GATC Biotech (Konstanz, Germany).

Whole-genome shotgun sequencing yielded 136,345 raw reads with 614,329,727 bp of raw sequence. Assembly was completed with the Hierarchical Genome Assembly Process (HGAP) algorithm implemented in the PacBio SMRT Analysis software (Pacific Biosciences, Menlo Park, CA, USA) and subsequently upgraded by PBJelly (6). The assembly resulted in three contigs totaling 6,038,906 bp, with a maximum contig size of 5,624,359 bp (*N*_50_, 5,624,359 bp; *N*_90_, 260,105 bp), 101.7-fold overall coverage, and a G+C content of 45.23%.

The closest relative of Mc5Re-14 based on the full-length 16S rRNA gene sequence is *P. polymyxa* strain SC2 (accession no. NR_102803, 99% sequence similarity), a plant growth-promoting rhizobacterium with broad-spectrum antimicrobial activity (7). Digital DNA-DNA hybridization (DDH) using GGDC 2.0 (8–10) against the genome sequence of *P. polymyxa* strain SC2 (accession no. CP002213) estimated a DDH of 86.40% ± 2.44% confidence, indicating that they have 94.41% probability of being the same species and 59.38% probability of being the same subspecies.

Annotation was conducted on the RAST Web server using RAST gene calling based on FIGfam Release70 (11, 12), and additional annotation was completed on the BASys Web server using Glimmer gene prediction (13, 14). The genome annotation contained 5,433 predicted protein-coding genes, 109 tRNA and 42 rRNA loci, and 450 predicted SEED subsystem features.

The *P. polymyxa* strain Mc5Re-14 genome has broad functional potential and a novel prophage. The genome encodes synthases for mycosubtilin, bacitracin, tyrocidine, gramicidin, and plipastatin, as well as seven additional polyketide synthases. Mc5Re-14 revealed genes coding for chitinase A1 and extracellular glucanases and biosynthesis gene clusters for auxin and spermidine production. The prophage encoded by the Mc5Re-14 genome shares homology with phage structural proteins from the giant *Bacillus* siphophage vB_BanS-Tsamsa in *Bacillus anthracis* (15). The Mc5Re-14 genome revealed promising biocontrol and plant growth-promoting capabilities.

### Nucleotide sequence accession numbers.

This whole-genome shotgun project has been deposited in the European Nucleotide Archive under the accession no. CVPD00000000. The version described in this paper is the first version, CVPD01000000.
